# Medical student training with next-generation handheld ultrasound devices – hands on examination of fetal biometry in obstetrics

**DOI:** 10.1186/s12909-025-06683-0

**Published:** 2025-01-22

**Authors:** Ruben Plöger, Julia Matschl, Adeline Walter, Ulrich Gembruch, Brigitte Strizek, Charlotte Behning, Agnes Wittek, Florian Recker

**Affiliations:** 1 Department of Obstetrics and Prenatal Medicine, University Hospital Bonn, Venusberg Campus 1, Bonn, 53127 Germany; 2 Institute for Medical Biometry, Informatics and Epidemiology, University Hospital Bonn, Venusberg Campus 1, Bonn, 53127 Germany

**Keywords:** Fetal biometry, Obstetrics, Personal-device-based-point-of-care-ultrasound, Point-of-care-ultrasound, POCUS, Semiconductors, Piezo-based, Chip-based, Gynecology, OSCE, Medical training

## Abstract

**Introduction:**

The technical development of ultrasound devices based on silicon chips has revolutionized ultrasound examinations, leading to the implementation of these portable handheld devices (PUD) in different medical fields. However, training on these devices is necessary to assure appropriate use and ensure valid results. While training programs for the use of conventional standard ultrasound devices (SUD) have been described, no training program for these handheld devices has been developed thus far.

**Methods:**

A training program for obstetric ultrasound examination was modified through the addition of an extra module focusing on the use of these PUDs. After the module the students had to attend an OSCE in which their skills of using the PUD and the SUD were tested and analyzed by applying the agreement rate, the intraclass correlation coefficient (ICC) and Bland–Altman plots. Furthermore, the students’ ultrasound results were compared with those of trained physicians by employing the one-sample Student's t-test. After the OSCE, the students answered a survey regarding their experience and their expected use of these devices.

**Result:**

An agreement of one hundred percent was reached for basic parameters such as fetal position, placental position, fetal heartbeat and for the classification of the amniotic fluid. The ICC showed a good to moderate agreement between the results of fetal biometry achieved by SUD and PUD. The T-test results confirmed a high reliability between the physicians’ results and the students’ results, independent of the used device. The students remarked a good handling of the ultrasound devices and supported the use in their future specialties.

**Discussion:**

The reliability between the examinations using the SUD and PUD were high but lower than the results observed for trained physicians. Therefore, the implementation of an additional module for portable ultrasound teaches the students to reliably examine basic obstetric parameters and provides a solid basis for further training and improvement of ultrasound skills in use of PUD.

**Supplementary Information:**

The online version contains supplementary material available at 10.1186/s12909-025-06683-0.

## Introduction

A new generation of portable ultrasound devices (PUD) based on silicon chips consists of a portable ultrasound probe connected to screens such as a cell phone or tablet. In contrast to conventional standard ultrasound devices (SUD), with their need for a cart and electricity, a quick and universal application of the PUD allows point-of-care ultrasound in various areas. In obstetrics, their use in identifying complications before, during, and after delivery has been demonstrated [1]. The accuracy of these devices has been well-proven in the case of fetal biometrics, amniotic fluid assessment and uterus examination after delivery [2–7]. However, in these studies, only experienced ultrasound examiners conducted the examinations, raising into question the need for training of less-experienced ultrasonographers, such as students, in the use of these next-generation ultrasound devices in obstetrics.

Medical students [8, 9] and residents [10, 11] often consider their ultrasound training to be insufficient. As an approach to improve their ultrasound training, courses [12, 13] and comprehensive ultrasound curricula for medical students [14] and residents [15] have been developed and implemented, whereby objective structured clinical examinations (OSCE) are often chosen to test the progress [16]. However, teaching of obstetric ultrasound examinations using the PUD has not been reported thus far. This study now presents an approach for teaching obstetric ultrasound examinations with a PUD. To gauge the success of the teaching method, the results of the students’ examinations using PUD and SUD were compared and analyzed in relation to the results of trained physicians. The medical students’ self-assessment and their feedback regarding the PUD were evaluated using a Likert scala.

## Methods

**Fig. 1:**
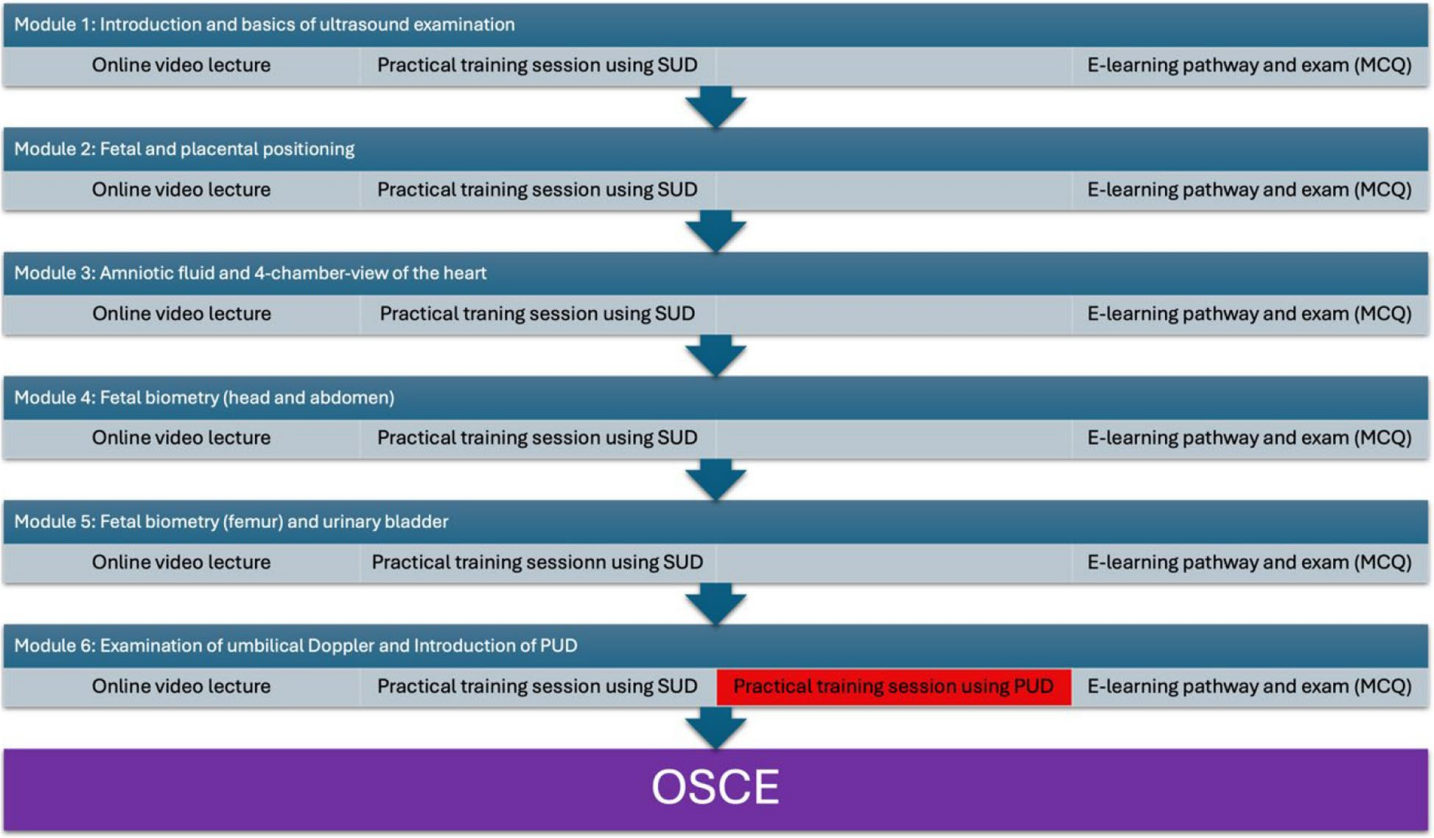
Overview of the training program of the students. MCQ: multiple choice question, OSCE: objective structured clinical examination, SUD: standard ultrasound device, PUD: portable ultrasound device

A prospective investigation regarding a new training program for medical students was performed. The study was approved by the University Hospital Bonn's Ethics Board (No. 269/23-EP). A training program was developed and implemented in the curriculum as a voluntary elective based on the guideline for reporting evidence-based practice educational interventions and teaching (GREET, [17]). The GREET checklist is presented in Table 1. In winter 2023/2024 15 medical students completed the program consisting of five modules, which was supplemented with a module regarding the use of a PUD. The medical students were randomly chosen from a pool of applicants and signed confirmed consent. In every module they attended an online video lecture, a practical training session with patients and an E-learning pathway with a final exam using multiple choice questions (s. Fig. 1). The five modules focused on the following topics: ultrasound basics (hand position and settings), examination of fetal and placental positioning, presentation of the urinary bladder, amniotic fluid assessment, presentation of a 4-chamber-view of the heart to determine heart activity, and examination of fetal biometry and the umbilical artery Doppler signal. All topics were in alignment with both the ISUOG recommendations [18] and the DEGUM level 1 standards [19, 20]. Up until the final module the medical students were trained solely using the SUD (Voluson E10 and GE Voluson S8 by General Electric, Boston, Massachusetts, USA), while in the additional sixth module they were introduced to the PUD (Butterfly iQ by Butterfly Network, Guilford, Connecticut, USA) and could practice its application. The modules took place every two weeks. At the end of the program an OSCE [21] was realized: The medical students had to perform an obstetric ultrasound examination using a SUD and a PUD, in a randomly assigned order. For the OSCE, five patients were asked to participate in the examination. All of these third-trimester patients were healthy but hospitalized due to fetal or pregnancy-related complications such as growth restriction. They signed informed consent and agreed to the analysis of the results. The patients were examined by a trained physician with more than five years’ experience (BS and UG). The results of the fetal biometry, including the estimated fetal weights (EFW) calculated by Hadlock [22], are presented in Table 2. The students were assigned alphabetically to a test date and thus randomly to a patient. The students had 20 min to examine the patient using the SUD and PUD in front of the examiners (RP, JM, AW and FR). Thereby, they had to show cardiac activity, fetal presentation, placental location and amniotic fluid assessment. Furthermore, they had to perform a fetal biometry and measure the single-deepest amniotic fluid pocket.

**Table 1:** GREET checklist: PUD: portable ultrasound devices, SUD: standard ultrasound devices, OSCE: Objective structured clinical examination

INTERVENTION	Implementing the use of portable ultrasound devices (PUD) in an educational program of obstetric ultrasound
THEORY	Improving medical training and patient care through additional training with PUD alongside the established seminar for obstetric ultrasound with standard ultrasound devices (SUD)
LEARNING OBJECTIVES	Performing an examination of fetal and placental positioning, amniotic fluid assessment, presentation of a 4-chamber-view of the heart to determine heard activity and of the urinary bladder, examination of fetal biometry and the umbilical Doppler with a PUD
CONTENT	Need for an examination of a pregnant woman without the availability of SUDs such as in situations outside of the hospital
MATERIALS	SUD: Voluson E10 and GE Voluson S8 by General Electric, Boston, Massachusetts, USA PUD: Butterfly iQ by Butterfly Network, Guilford, Connecticut, USA Patient bench
EDUCATIONAL STRATEGIES	online video lecture, a practical training session and an E-learning pathway
INCENTIVES	Learning ultrasound diagnostics
INSTRUCTORS	Experienced ultrasound examiners
DELIVERY	Face-to-face teaching in groups with practical training (ratio of learner to instructors: 15–4), online video lectures, E-learning pathway
ENVIRONMENT	Hospital ward
SCHEDULE	Final module (6th)
TIME	Two hours in group teaching, unlimited access to the online learning package
Specific adaptation	No specific adaptation
MODIFICATION	No modification during the course
ATTENDANCE	Using an OSCE to assess the knowledge and performance
IMPLEMENTATION	No change during implementation

**Table 2:** Overview of the fetal biometry and single deepest pocket of the five patients, measured by trained ultrasound physicians using standard ultrasound devices. BPD: biparietal diameter, HC: head circumference, AC: abdominal circumference, FL: femur length, EFW: estimated fetal weight, SDP: single deepest pocket, Min: minimum, Max: maximum. Gestational age is stated in weeks and days, BPD, HC, AC, FL and SDP is stated in centimeter, EFW in g

	Gestational Age	BPD	HC	AC	FL	EFW	SDP
Patient 1	29 + 3	7.76	26.87	21.9	5.37	1104	7.61
Patient 2	34 + 6	8.98	31.87	32.52	6.82	2826	5.66
Patient 3	29 + 4	7.34	26.82	25.45	5.44	1369	4.26
Patient 4	36 + 5	9.65	33.45	35.97	7.31	3695	5.00
Patient 5	32 + 3	8.14	28.87	28.17	5.77	1802	7.41

A survey based on Likert scale regarding the use of PUD was developed for this study. After the OSCE exam the medical students completed the survey and submitted the survey letter anonymously in a box. The surveys were analyzed following the analysis of the OSCE results.

### Statistical analysis

Statistical analyses and graphical representation were conducted using Excel and PowerPoint of the Microsoft office package (Microsoft, Redmond, WA, USA) and the Statistical Package for the Social Sciences (SPSS) software, version 27 (IBM Corp., Armonk, NY, USA). The sample size was limited to 15 students due to practical feasibility. To evaluate the sonographic results determined via SUD and PUD, the intraclass correlation coefficient (ICC) employing a two-way random-effect, agreement model with a 95% confidence interval and Bland–Altman plots [15] were applied. An ICC value of 1.0 indicates a high degree of agreement whereas a value of 0 indicates a low degree of agreement [23]. Negative values are be interpreted as zero. This is known to appear in the case of a small sample size [24]. For the one-sample student’s t-test the null hypothesis stated that the results of the students using either the SUD or the PUD would not differ from the results of the trained physicians. The differences of the trained physicians’ results and the students’ results using a SUD as well as the differences of the trained physicians’ results using a SUD and of the students’ results using a PUD were calculated (delta). Next, mean and standard deviation were measured. These two Delta-values were compared using a one-sample student’s t-test. The significance level was set at 5%.

## Results

**Fig. 2:**
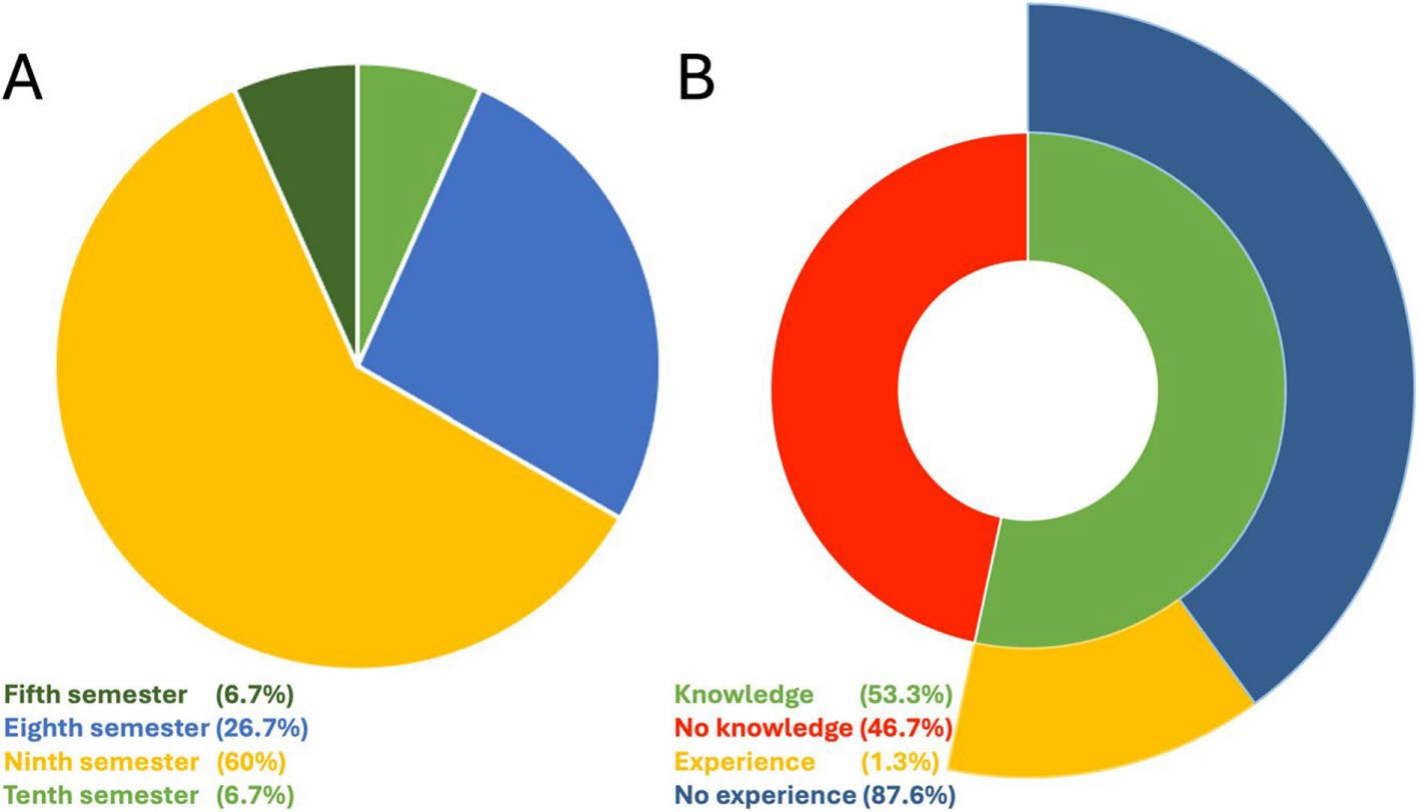
Pie chart presenting the students clinical semester (**a**) and a sunburst chart presenting the prior knowledge of the students regarding the existence and use of portable ultrasound devices (**b**). Pie chart presentation: students in the fifth semester (dark green), in the tenth semester (bright green), in eighth semester (bright blue) and in the ninth semester (yellow). Sunburst chart: no knowledge about the portable ultrasound devices (red), prior knowledge (bright green), previous use of portable ultrasound devices (yellow), no experience (dark blue)

All of the 15 chosen medical students completed the module and participated in the OSCE using a SUD and a PUD, so that all parameters could be collected. Regarding the characteristics of the medical students, 86.67% of them were female and 13.37% of them were male. Further characteristics are presented in Fig. 2.

Patients 1,2 and 5 were examined by four students, patient 4 was examined by two students, and patient 3 was examined by one student. The fetal biometrics of these patients obtained by the trained physician are presented in Table 2.

The comparison of cardiac activity, fetal presentation, placental location and amniotic fluid classification between the examinations using SUD and PUD yielded an agreement rate of one hundred percent. In these categories the results of the medical students agreed one hundred percent with those of the trained physicians.

**Fig. 3:**
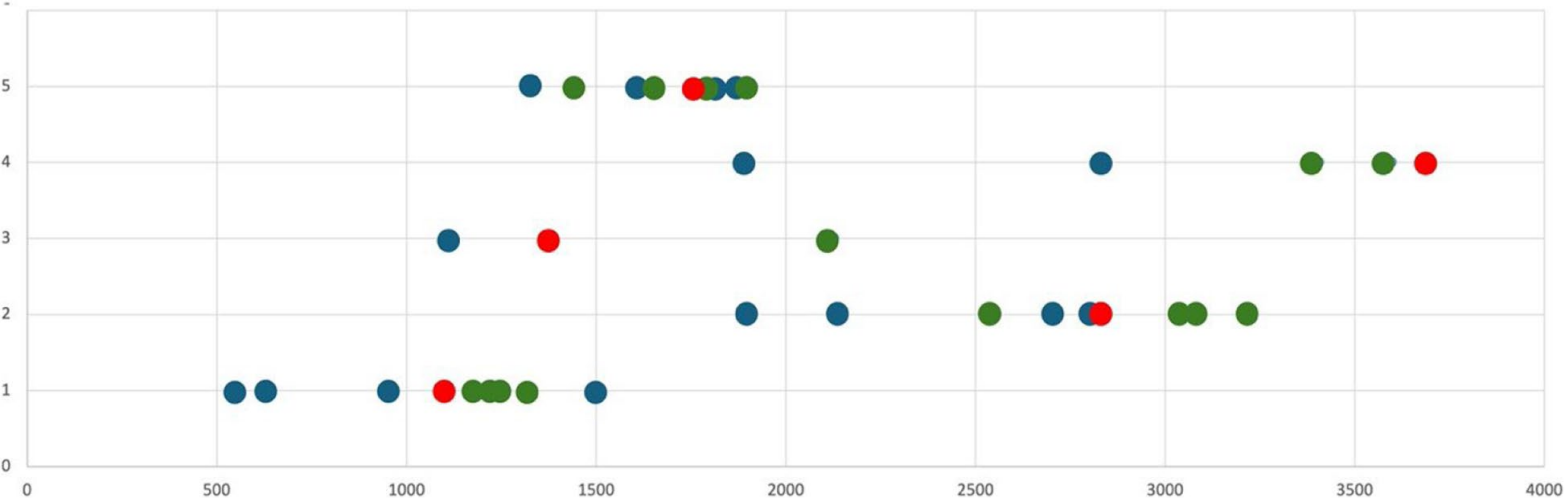
Illustration of the estimated fetal weight of the five examined fetuses based on the fetal biometries of the students using standard ultrasound devices (SUD, green) and portable ultrasound devices (PUD blue), and of the physician trained in ultrasound diagnostics (red) using SUD

Overall, the EFWs determined using the SUD were closer to those measured by the trained physician than the mean EFWs determined using the PUD (s. Fig. 3).

**Fig. 4:**
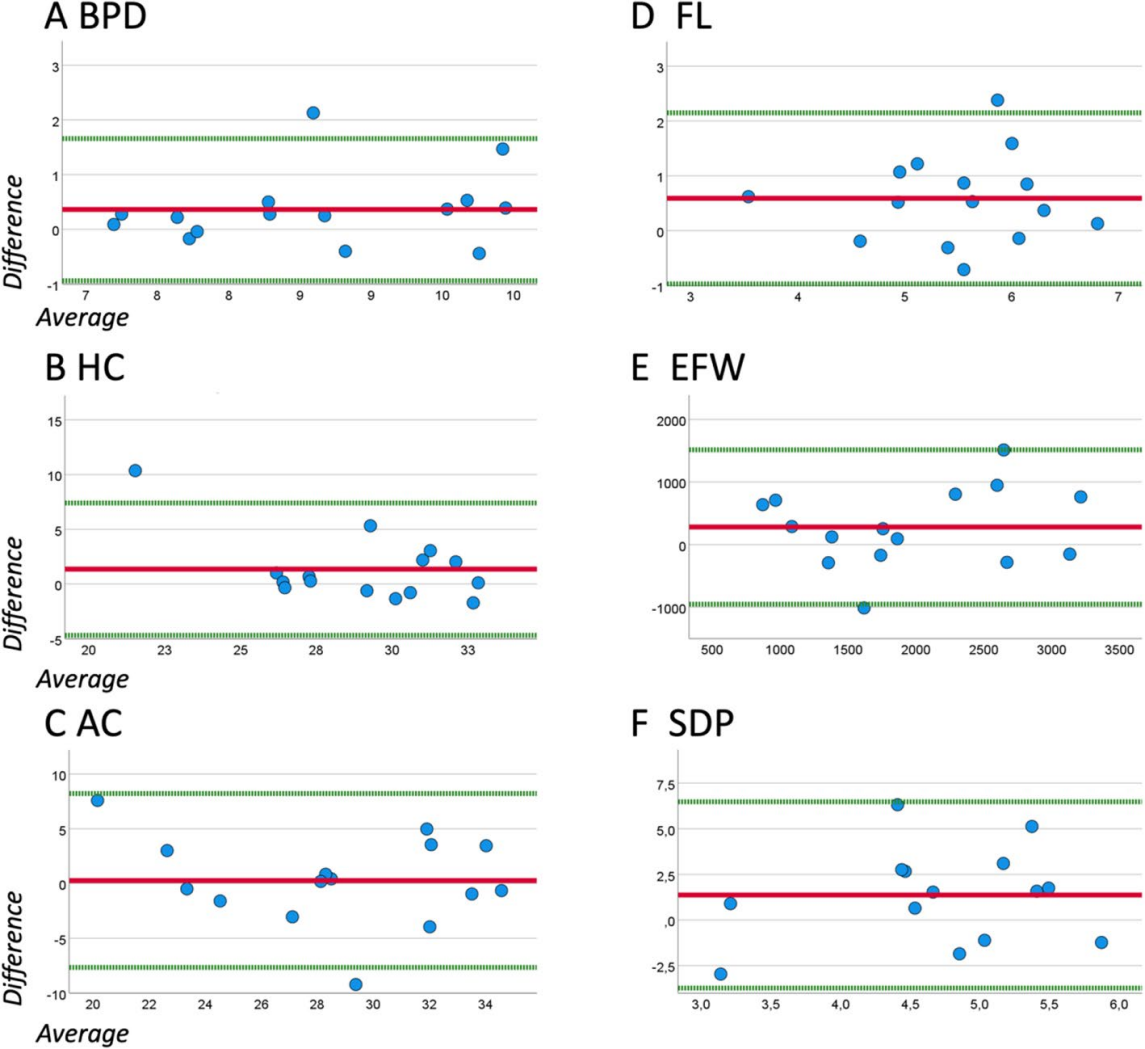
Bland–Altman-Plot to analyze the agreement between the students’ results using standard and portable ultrasound device for biparietal diameter (BPD, A), head circumference (HC, B), abdominal circumference (AC, C), femur length (FL, D), estimated fetal weight (EFW, E) and single deepest pocket (SDP, F): Upper and lower limits of 95%-agreement depicted by the dotted green line and the mean difference is shown by the red continuous line. The abscissa shows the average of the measurement using a PUD and a SUD whereas the ordinate shows the difference between both results. The difference and average are stated in cm (A-D, F) and in g (E)

The ICC values (Table 3) indicated a good agreement for the measurements of the BPD, AC and EFW, a moderate agreement for the measurements of the HC and FL and a poor agreement for the measurements of the SDP [23]. The Bland–Altman analysis for BPD, HC, AC, FL, EFW and SDP demonstrated an average difference of 0.36 cm, 1.36 cm, 0.28 cm, 0.59 cm, 281.67 g and 1.37 respectively. The 95% limits of agreement were between −0.94 cm to 1.67 cm, −4.69 cm to 7.41 cm, −7.66 cm to 8.22 cm, −0.97 cm to 2.15 cm, −952.16 g to 1515.50 g and −3.73 to 6.48 respectively (Fig. 4). The scatter analysis of all Bland–Altman plots revealed a random pattern indicating no bias. The t-test (Table 4) indicated that certain test results were too extreme to be explained by the standard deviation: this was observed in the case of the BPD and FL measured with the SUD, and of the FL, EFW and SDP measured with the PUD. Significantly different results with a large effect size were obtained in the cases of the BPD measurements using SUD and of the FL and SDP measurements using the PUD. Significant differences with medium effect sizes were found for the FL using SUD and of the EFW using PUD. For all other parameters the deviation from the standard value lacked significance so that the null hypothesis is assumed to be true, confirming that the ultrasound devices have no impact on the examination results.

**Table 3:** Intraclass correlation (ICC) coefficient with coincidence interval (95%) for measurements (fetal biometry and single deepest pocket) obtained by the medical students with the portable ultrasound device (PUD) in comparison to the standard ultrasound device (SUD)

	ICC (CI 95%)
Biparietal diameter (BPD)	0.861 (0.562 – 0.954)
Head circumference (HC)	0.727 (0.236 – 0.906)
Abdominal circumference (AC)	0.803 (0.400 – 0.934)
Femur length (FL)	0.672 (0.029 – 0.890)
Estimated fetal weight (EFW)	0.814 (0.463 – 0.937)
Single deepest pocket (SDP)	−1.084 (−4.056 – 0.320)

**Table 4:** Descriptive statistics and T-test of the difference (delta-value) between the results of the trained physicians with the standard ultrasound device (SUD) and of the students with the SUD and the portable ultrasound device (PUD), respectively: BPD: biparietal diameter, HC: head circumference, AC: abdominal circumference, FL: femur length, EFW: estimated fetal weight, SDP: single deepest pocket, SD: Standard deviation, df: degree of freedom

		BPD (cm)	HC (cm)	AC (cm)	FL (cm)	EFW (g)	SDP (cm)
SUD	Mean	−0.37	−0.08	−0.31	0.58	22.87	0.82
	SD	0.12	0.72	1.56	0.78	210.94	0.62
	T score	−3.23	−0.44	−0.76	2.89	0.42	1.32
	df	14	14	14	14	14	13
	P value	0.01	0.67	0.46	0.01	0.681	0.21
	Cohen’s d	0.835	−0.12	−0.197	0.75	0.11	0.35
PUD	Mean	−0.01	1.34	−0.03	0.92	392.60	2.42
	SD	0.62	3.28	3.69	0.95	625.44	1.70
	T score	−0.83	1.58	−0.03	3.76	2.43	5.55
	df	14	14	14	14	14	14
	P value	0.94	0.14	0.98	0.01	0.03	0.00
	Cohen’s d	−0.02	0.41	−0.01	0.97	0.63	1.43

**Fig. 5:**
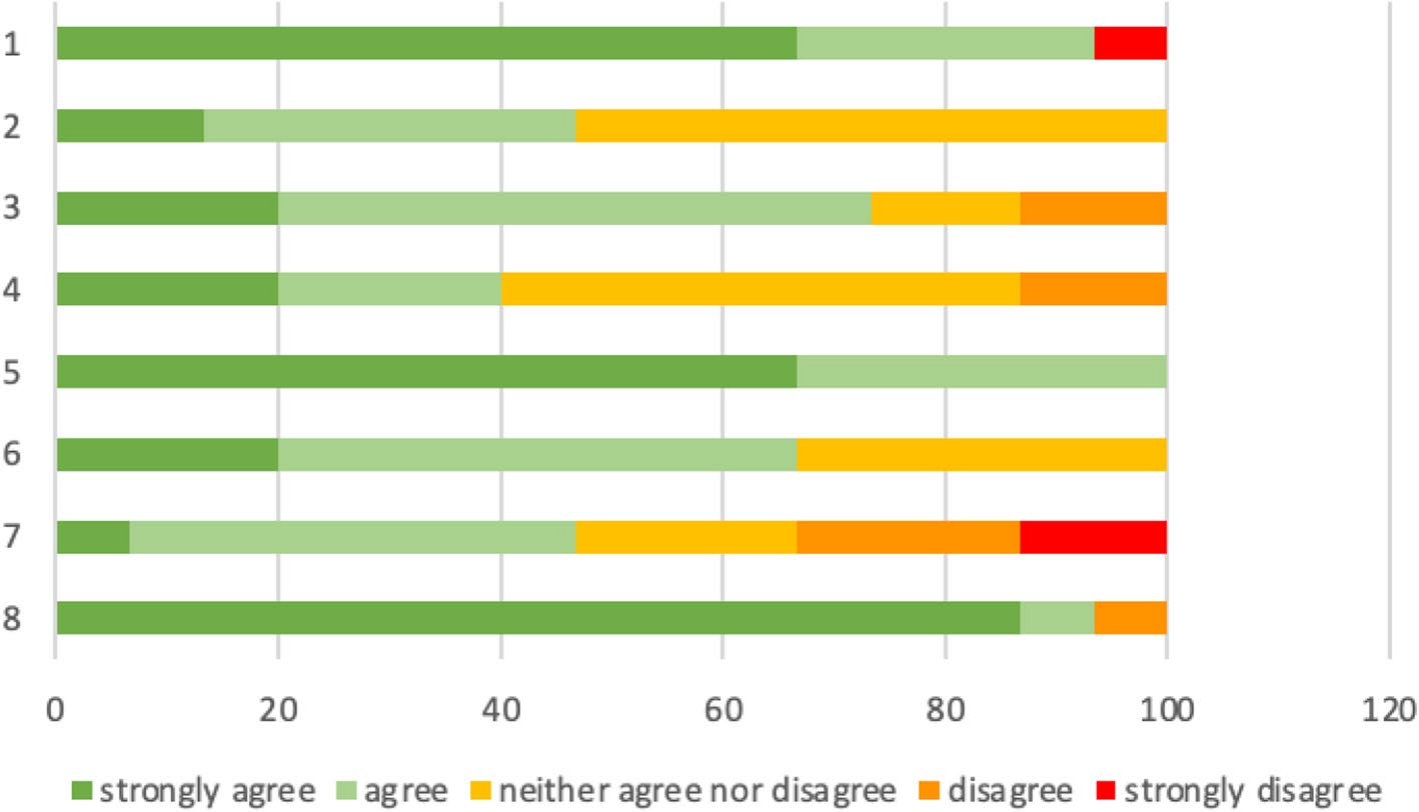
Bar chart presenting answers using Likert scale about the portable ultrasound device (PUD): The five response options range from “strongly agree” (dark green) to “strongly disagree” (red). The statements are as followed: (1) I found the operation of the PUD intuitive. (2) I was able to solve the task with PUD just as well as with the SUD. (3) I felt confident using the PUD. (4) I came to the same results with the PUD as with the SUD. (5) I can imagine using the PUD in everyday clinical practice. (6) I am confident in the future use of the PUD. (7) The PUD will influence my choice of a specialization. (8) I can imagine further applications of the PUD outside obstetrics. The approval rate of the students is presented as a percentage on the abscissa

The survey using Likert scale was completed by all 15 medical students and the results are presented in Fig. 5.

## Discussion

An examination with a PUD was implemented as part of an obstetric ultrasound training program for medical students. The medical students’ assessments of fetal cardiac activity, fetal presentation and placental position using the PUD and SUD had a high agreement rate. A similarly high agreement rate was seen when comparing the students’ results with those of the trained physicians using a SUD. These high assessment rates are in harmony with the existing studies of PUD’s reliability [2, 4, 5]. Therefore, this basic obstetric assessment seems to be secure and quickly learnable with a PUD.

The students’ measurement of the fetal biometry with the PUD displayed a good to moderate agreement in comparison to the SUD, with exception of the measurements of the SDP. The ICCs of the biometric variables in this study lacked an excellent agreement, in contrast to the known accuracy and reliability studies of PUD used by well-trained ultrasound examiners [2–7], of which some coincidently used the same device as in this study [2–4, 7]. Therefore device-specific bias can be ruled out. The suboptimal ICCs may be caused by the limited experience of the medical students in use of the PUDs. Even some professional examiners show only moderate agreement (0.66) as reported for the FL in one study [5], and are able to improve their ultrasound skills by regular clinical training [25]. Thus, the good to moderate agreement in this study can be counted as a success regarding the short teaching time of only one module. Follow up studies would help determine whether and when this discrepancy disappears and thus how much training is necessary. The implementation of PUD in other disciplines such as dermatology [26] or internal medicine [27] further supports the good transferability of the examination technique from a SUD toward the PUD. The wide range of the confidence interval of the ICCs and limits of agreement of the Bland–Altman plot reflects the individual learning success or/and the low number of participants in comparison to previous studies regarding well-trained ultrasound examiners (number of participants: 45–100) [2, 4–6]. A future study with an increased number of students may help to see the influence on the confidence interval of the ICC and the limits of agreement of the Bland–Altman plot. The students’ t-test was performed and revealed a high reliability between the physicians’ results and the students’ results regardless of their use of the PUD and SUD for almost every parameter. The medical students using the PUD achieved similar BPD, HC and AC measurements as the trained physicians but reached significantly different results of the measured FL and the calculated EFW – maybe caused by the influence of the FL in the EFW Formula [22]. The effect size of the difference regarding the FL is larger when using the PUD than when using the SUD, which may explain the still comparable result for the EFW between the trained physicians and the students using the SUD. However, the medical students using SUD measured significantly different results for BPD and FL in comparison to the trained physicians, highlighting the difficulty of these parameters. The different result of the SDP correlates with the poor ICC published in a study focusing on well-trained ultrasound examiners [5]. This may be caused by changes in the fetal position or measurements from different positions. The high variance in SDP assessment had no effect on the final clinical diagnosis of normal hydramnios, oligohydramnios or polyhydramnios, whose classification with the PUD were the same as with the SUD in all cases.

The answers of the survey after the OSCE are in harmony with the good to moderate ICC: The students gave high approval scores in their self-assessment in the use of both ultrasound devices and in the comparability of their results. This reflects the statistical analysis of the results and supports the quality of the developed training program. The medical students’ approval about the intuitive use of the PUD supports the high agreement rates and moderate to good ICCs despite their limited amount of training with the PUD and argues for an easy transfer of knowledge and skills from the SUD toward the use of the PUD. Furthermore, the students express confidence about applying the PUD again in the future. Regarding the PUD in general the highest agreement rates in the survey were detected for the statements: “I can imagine using the PUD in everyday clinical practice.” and “I can imagine further applications of the PUD outside obstetrics” and thus indicate the medical students’ vision of PUD’s use. The PUD could be integrated more widely into medical education: (1) The low cost of the PUD could enable the individual equipment of students with these ultrasound devices and thus further implement comprehensive ultrasound curriculum for medical students [14]. (2) The portability of PUD allows ultrasound training via teleguidance and is thus feasible via online teaching, as was necessary during the COVID-19 pandemic [28, 29], and allows teaching outside of ultrasound diagnostic centers which often reach capacity due to patient care. Therefore, the PUD may solve the problem of inadequate ultrasound education [8]. However, this training module and the use of PUD did not have an impact on the medical students’ decision for a medical specialization in one third of the cases, while in 46% of medical students did see an effect on their decision emphasizing the importance of elective courses during medical training and PUD’s relevance in future medical work.

Several limitations must be considered to interpret the results of this study. The sample size of 15 medical students is not representative, especially because they were chosen from an application list. Based on voluntary participation in the course only medical students with a high interest, commitment and motivation attended and therefore may skew the results. The fact that two of them had previous experience using a PUD in other medical disciplines could lead to a discrepancy regarding the level of expertise, although these students’ results were ultimately similar to those of the other medical students. An increase in the number of participants would result in less hands-on experience per medical student due to the limited time of patients and the teaching physicians. Time limitations for patients further led to unbalanced distribution between patients and students. Incorporating simulation-based assessments [30] for fetal biometry such as the established 3D-printed nuchal translucency model [31] would standardize the conditions in regard to the fetal positions and maternal body composition. While the different initial conditions make a comparison of the results more difficult, they also mirror the real-world scenarios students will encounter in clinical practice. The fact that the students only had 20 min to perform the examination using the PUD and the SUD in an examination scenario simulates the examination time during shifts in a hospital and thus also reflects real-world scenarios. The data collection during an OSCE puts pressure on the students. The argument for this examination setup was to motivate the students to achieve their best results. The study's focus was also somewhat constrained, centering on basic obstetric ultrasound parameters and categorical variables such as cardiac activity, fetal presentation, placental location, and amniotic fluid volume. Expanding the range of examined variables to include a more comprehensive set of fetal biometry and maternal factors could offer a more detailed understanding of PUDs’ capabilities and limitations. The results of this study are specific for one portable ultrasound system with a non-piezo, chip-based technology of one manufacturer, whereby almost every ultrasound manufacturer has developed their own portable, handheld ultrasound device, their own technical specifications and their own different platforms and apps.

## Conclusion

This study shows a good transferability of the examination technique from a SUD towards the examination with a PUD. This impression needs further testing in larger cohorts and in more standardized conditions until similar real-life scenarios can tested. The positive feedback of students supports the advantages of portable ultrasound technology and heralds a new era in obstetric ultrasound education and diagnostic. These inspiring results may lead to comparing further handheld devices and implementations of portable ultrasound devices in other medical specialties either during the daily routine or during teaching.

## Supplementary Information

Supplementary Material 1.

## Data Availability

Data available on request from the authors.
