# Supplementary figures and images for: Medical student training with next-generation handheld ultrasound devices – hands on examination of fetal biometry in obstetrics
Source: BMC Med Educ. 2025 Jan 22;25:103. doi: 10.1186/s12909-025-06683-0 (PMC11752848; doi:10.1186/s12909-025-06683-0)

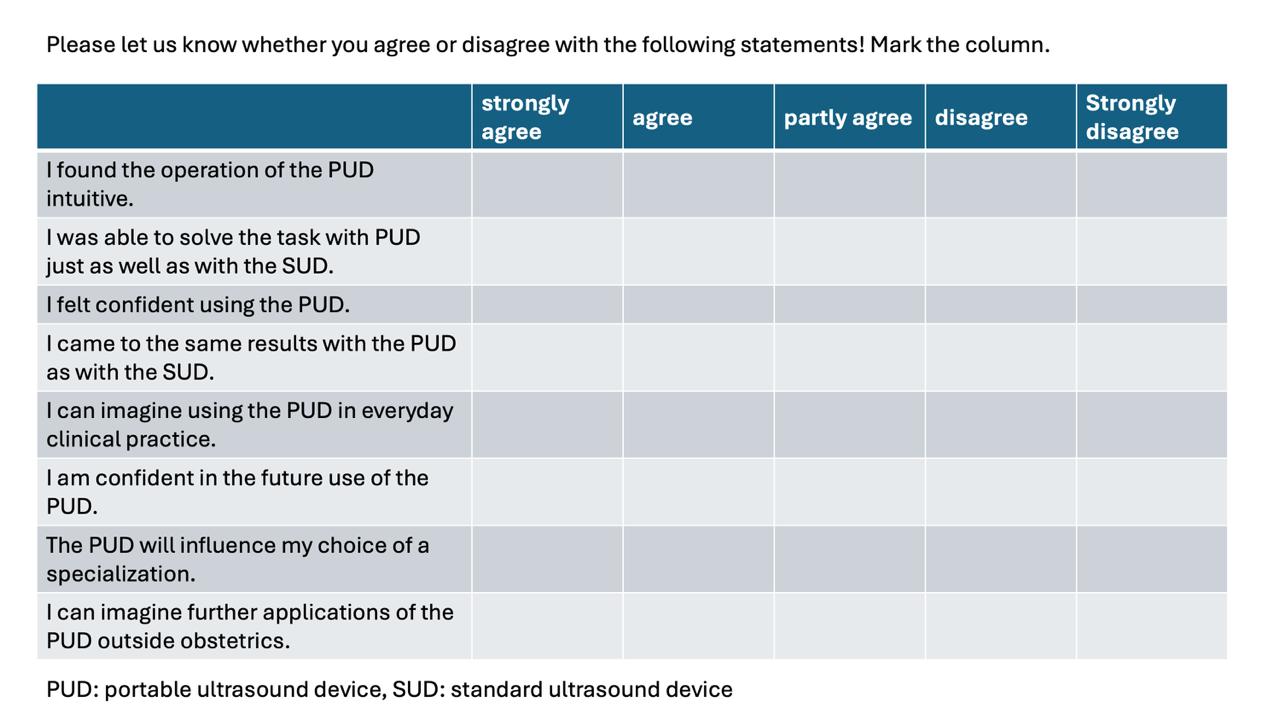

Supplement: Supplementary file 1 — Supplementary Material 1. [file 12909_2025_6683_MOESM1_ESM.jpeg]
